# Brain Surgery

**Published:** 1888-03

**Authors:** 


### BRAIN SURGERY.

Since Molius trepanned Prince Rupert's skull, brain surgery has always had a fascination for English surgeons. No doubt many timorous members of the craft of surgery from time to time protest against and prophecy evil of trephining, particularly so when the operation is intended merely as a preliminary to a more serious step. Percival Pott tells us how in his day a simple puncturing of the great longitudinal sinus, which was productive of the happiest effects, brought upon him many and grave censures. Yet Pott's example was not lost; our great Irish surgeon, O'Halloran, became noted for his daring and success in brain surgery, and later still,Guillaume Dupuytren shocked the timorous and encouraged the thoughtful by evacuating a cerebral abscess. Nevertheless, brain surgery became a forbidden thing, not because there was extreme diffiulty in its being done, nor from want of success in its performance, but because of the incongruous admixture of theology with surgery, and the delimitation of the cerebral structure from the domain of surgery—it being the soul centre. And although broken skulls had allowed cerebral matter to protrude, and when protruded the objection to surgical interference was not urged, still, for a surgeon to contemplate opening the sacred inclosure of the skull with a trephine for the purpose of evacuating diseased products was condemned, though a brickbat might smash the skull, and healthy brain tissue be extruded and excised without anything like the same shock to public sensitiveness. The fact that from time immemorial a cleric had with a sharp stone scraped a hole in children's skulls to liberate the demon that caused the convulsion which to-day we ascribe usually to teething, was either forgotten or explained away as part of a religious ceremony now happily discontinued. Nevertheless, medicine in the face of all these difficulties made progress. The observation that the brain was liable to disease and that nerve tissue was as directly subject to material injuries gradually gained acceptance. Pressure as from a blood-clot was found to as completely stay its functional activity as it would that of the lung, and purulent collections were found to influence it both by pressure effects and inflammatory effects much as they do other tissues. More careful examinations of the cerebral lesions soon detected the fact that pressure in certain regions was found associated with distinct symptoms. Notably the effect produced by pressure on the internal gyration of the left frontal lobe. Everything was telling of a young generation's dissatisfaction with the "rest and be thankful" of their immediate predecessors, and when Dr. Ferrier's experiments told that the obscurity which enveloped psychology was to terminate in the brilliancy of natural light the thoughtful truthseekers rejoiced that man's intelligence had at last enabled him to contend with a hope of success against diseases which, by attacking his organ of mind, had deprived him of his prerogative of the "thinking" faculty, and reduced him to the level of the brute. To Mr. Horsley is fairly due the credit of first practically demonstrating the utility of Dr. Ferrier's experiments, and as might be naturally expected his example soon found many followers. Even the merest beginner was anxious to try his success in this new domain that medicine had reclaimed. The topography of the cerebrum became the most fascinating of studies, and the localization of the functions of thought and intellectual expression now were counted as fairly within the physiologist's domain, and as a consequence demanded the therapeutist's and the surgeon's study. Beaconsfield's maxim, however, holds good, and the case that recently occupied the Medical Section of the Royal Academy of Medicine, Ireland, about which there has been so much discussion in our columns, exemplifies it. We cannot, however, expect men trained in the past to devote the same care to the study of newly developed sciences that they did in their student days, nor is it reasonable to expect that they could divest themselves of the bias of their early training. Still, in a rapidly progressive science like surgery, dependence on the rejected formula of a dead past can bring nothing but failure.—*Medical Press and Circular.*

